# Assessment and management of a 1.77‐cm^2^ mitral leaflet perforation as a subclinical cause of mitral regurgitation

**DOI:** 10.1002/ccr3.1722

**Published:** 2018-08-22

**Authors:** Paras Patel, Kevin Lee, Adedoyin Aderinto, Michael Benz, Antonios Tsompanidis

**Affiliations:** ^1^ Rowan University School of Osteopathic Medicine Stratford New Jersey; ^2^ Department of Anesthesiology The University of Toledo Medical Center Toledo Ohio; ^3^ Department of Cardiology, CarePoint Health Christ Hospital Jersey City New Jersey; ^4^ Family Medicine Residency Program, CarePoint Health Christ Hospital Jersey City New Jersey

**Keywords:** 2D echocardiography, anterior mitral leaflet, atrial fibrillation, color doppler, dukes criteria, infective endocarditis, mitral regurgitation, transesophageal echocardiogram

## Abstract

Anterior mitral valve perforations are commonly due to underlying infective endocarditis, which can lead to significant mitral valve insufficiency. In these cases, clinicians should have a high index of suspicion for infective endocarditis. After appropriate imaging, immediate surgical intervention is unequivocally the choice of management here for optimal patient outcomes.

## 1 INTRODUCTION

Mitral valve regurgitation (MVR) is most commonly caused by mitral valve prolapse, affecting 2%‐3% of the population.^1^ However, perforation of the anterior mitral leaflet (AML) is a rare and often underlooked organic cause of MVR. Infective endocarditis (IE) is attributable to almost one‐third of mitral valve (MV) perforation cases.^2^ Leaflet damage associated with vegetations is the primary mechanism for which weakened tissue and subsequent valve destruction occurs leading to pulmonary edema. Patients develop symptoms consistent with acute MVR and can develop new‐onset arrhythmias. Prior to diagnosing an AML perforation with echocardiography, it is essential to consider underlying etiologies such as IE, myxomatous degeneration, rheumatic fever, and mitral annular dilation. It is therefore important because early detection of an AML perforation necessitates valve reconstruction over conservative measures. We highlight a unique case and management of anterior MVR in which the patient's history and echocardiograph findings suggest subclinical endocarditis as being the underlying etiology despite not meeting Dukes criteria. Additionally, management of new‐onset atrial fibrillation (AF) in this patient contributed to the unique management of this case.

## 2 CASE HISTORY/EXAMINATION

**Figure 1:**
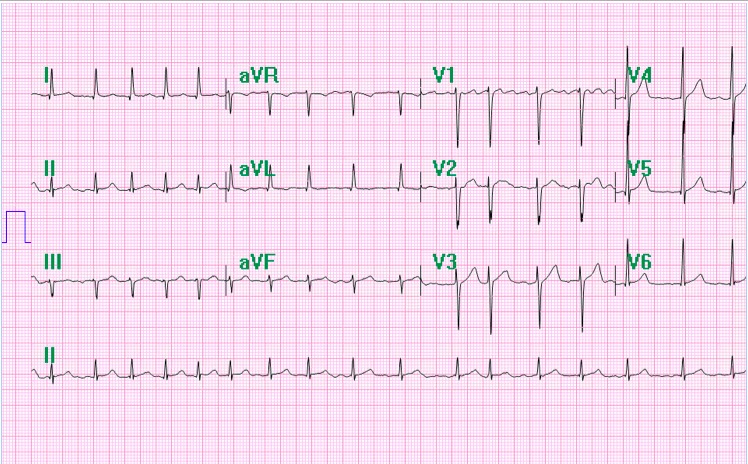
Atrial fibrillation with rapid ventricular response at the time of presenting symptoms

A 65‐year‐old incarcerated African American male with a past medical history of AF, mitral regurgitation, congestive heart failure with reduced ejection fraction (HF_r_EF), hypertension, diabetes, renal cancer, asthma, HIV, chronic hepatitis C, and IV drug usage presented to the emergency department with mid to lower left sternal chest pain that was squeezing in nature. His symptomatology was intermittent and associated with shortness of breath and a productive cough without hemoptysis or fever. The patient was initially evaluated in the emergency department. One of the studies performed was an electrocardiogram, which showed new‐onset AF with rapid variable ventricular response, as seen in Figure 1.

Furthermore, the patient was found to have troponin 0.06 ng/mL × 3, an elevated BNP of 1170 pg/mL (normal: 0‐900), and bilateral small pleural effusions on chest X‐ray. Of note, his CD4+ count was 233 cells/mm^3^, HIV viral load was 49 000 copies per mL of blood, and blood cultures showed no growth for organisms after 5 days. As part of the initial management, the patient was administered prophylactic aspirin, oxygen, and was evaluated with 2D echocardiography, revealing moderate mitral regurgitation during systole and a reduced forward ejection fraction of 35%‐40% with left ventricular global hypokinesia. The mitral regurgitation was classified as moderate based on a vena contracta of 0.45 cm.^3^ The 2D echocardiography also showed a moderately dilated left atrium (51.6 mm), as defined by the American Society of Echocardiography. Finally, it revealed a discontinuity of the interventricular septum suggestive of a ventricular septal defect. A coronary catheterization was also performed demonstrating normal coronary arteries and moderately depressed left ventricular systolic function with mitral regurgitation consistent with the 2D echocardiography results. In both imaging modalities, no signs of cardiac vegetations were noted. The results of this patient's workup supported evidence of mitral regurgitation and, more specifically, its acute exacerbation as reflected by his sudden symptomology.

**Figure 2:**
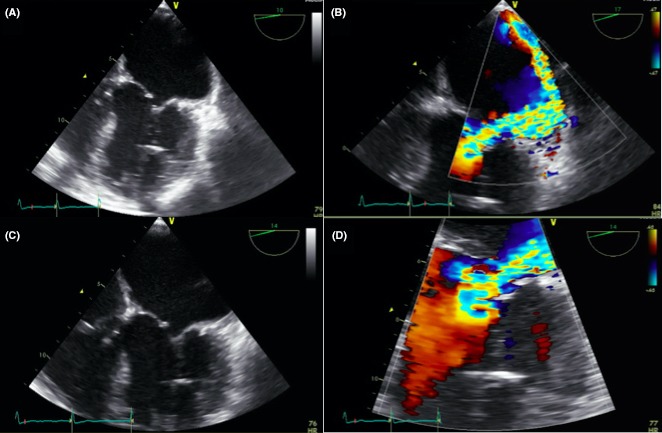
A, Transesophageal echocardiogram (TEE) of closed mitral valve. B, TEE with color flow Doppler image of A revealing severe mitral regurgitation traversing anterior mitral leaflet during valve closure. C, Transesophageal echocardiogram (TEE) of opened mitral valve. D, TEE with Color Doppler image of C revealing severe mitral regurgitation traversing anterior mitral leaflet during valve opening

**Figure 3:**
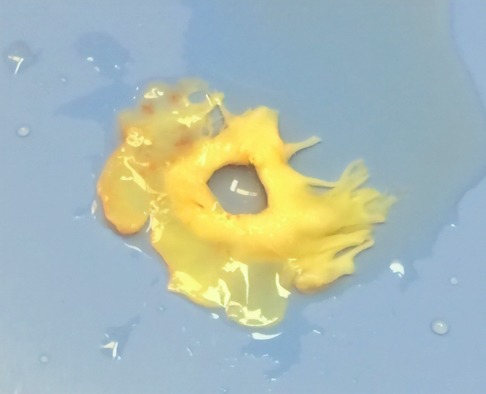
Gross specimen of perforated anterior mitral leaflet (AML) removed during surgery

2D echocardiography is able to capture blood flow traversing the MV during systole, which suggests a discontinuity within the valve itself, as opposed to an abnormal closure of the valve. Because of the limitations of 2D echocardiography, a transesophageal echocardiogram (TEE) was recommended as a follow‐up study, which can reveal leaflet discontinuities as small as 1 mm in resolution and distinguish those of anterior from posterior origin.^4^ TEE findings in this patient revealed an eccentric posterior jet mitral regurgitation in the AML as seen in Figure 2. This finding was confirmed by the gross specimen of the patients AML, which measured 2.8 × 2.5 × 0.30 cm in size and contained a perforation 1.77 cm^2^ in size, as seen in Figure 3. The gross specimen revealed no intracardiac abscess or histological signs of vegetations.

The severity of MVR was explained to the patient, and MV repair was recommended. The patient agreed with the plan and was transferred to another facility. However, because of the large size of the AML perforation, the patient was not an acceptable surgical candidate for valve repair. Instead, it was decided that total valve replacement would be more feasible and would improve overall mortality for the patient. A #27 Bovine Edwards MV was used as the replacement. After the operation for valve replacement, it was found that the patient became clinically unstable and his systolic blood pressure was reaching 180 mm Hg. The patient revealed a normal capillary refill time and stable heart rate; however, he had symptomatic complaints of chest pain and dyspnea. Immediate medical management necessitated acute stabilization of the patient's blood pressure and placement of chest tubes. Approximately 500 ccs of blood was collected in chest tubes prompting emergency surgical exploration of the mediastinum due to the extravasation of blood. After being stabilized in the Intensive Care Unit, the patient was discharged with medical management maintenance, including apixaban, losartan, aspirin, atorvastatin, furosemide, and metoprolol for his cardiovascular conditions and insulin aspart, tramadol, raltegravir, and emtricitabine/eenofovir for other medical conditions. While warfarin is the drug of choice for anticoagulation after prosthetic valve placement, the patient was prescribed Apixaban due to a history of poor follow‐up and similar outcomes between the two drugs.^5^ Nonetheless, he was still instructed to follow‐up with outpatient cardiology.

## 3 DISCUSSION

We present a unique case of MVR secondary to MV leaflet perforation, in which subclinical endocarditis was suspected and managed. During mitral regurgitation, the left ventricle allows a large amount of blood to be ejected backwards into the left atrium. This is appreciated as a drop in left ventricular systolic function and forward ejection fraction. In acute cases, there is a sudden increase in left atrial volume during ventricular systole due to an incompetent mitral valve. The normal left atrium is noncompliant, meaning it is not able to handle the sudden increase in left atrial pressure and volume that acute mitral regurgitation causes. This results in an increased left atrial pressure on echocardiography.^6^ As a consequence of acute regurgitation, there is an increase in pulmonary pressure and volume, resulting in pulmonary edema and ensuing shortness of breath.^6^ Due to pulmonary overload, myocardial voltage around the pulmonary veins is disrupted, and the patient also becomes prone to developing arrhythmias.^7^

Moreover, left atrial enlargement contributes to the generation of atrial re‐entry and AF, and progressive left atrial enlargement increases the risk of AF development.^7^ A recent study found that the underlying pathology seen in patients with MV disease was found to have elevated Type 1 collagen levels within left atrial appendage tissue, ultimately causing atrial remodeling predisposing the patient to possible development of AF.^8^ Identifying AF is essential in patients with mitral regurgitation because the heart already has a decreased ability to maintain cardiac output. In addition to the inherent thrombotic risk with the development of AF, elevated levels of immunoreactive vWF in the endocardial endothelium have been seen in patients with underlying mitral regurgitation, placing the patient at an even greater risk for developing intra‐arterial thrombogenesis.^9^ Therefore, immediate surgical intervention and long‐term oral anticoagulation are essential in managing these patients.

As mentioned, endocarditis is the leading cause of AML perforation. We suspect that the primary mechanisms that cause AML perforation in this patient include leaflet damage through infectious agents, endocarditis of the aortic valve, an aortic regurgitation jet disrupting the MV leaflet, or structural defects in mitral leaflets due to a prior IE.^4^ However, according to Dukes criteria, this patient did not fulfill the requirements for endocarditis. He did not meet the major or pathological criteria. On echocardiography and pathological examination, vegetation, abscess, or new valvular regurgitation was not found. Instead, the patient only fulfilled one minor criterion of IV drug use/predisposing heart condition. While this finding alone did not satisfy the requirements for possible endocarditis, echocardiography did show one of the known IE intracardiac complications—valvular perforation. Therefore, despite not meeting Dukes criteria, the patient's IV drug use and TEE findings should raise enough consideration for his AML perforation to be due to IE. The mechanism in which valve perforation occurs in patients with endocarditis can occur by primary vegetation destroying the valve tissue or secondary MV endocarditis. In secondary causes, an endocarditic aortic valve jet lesion or direct contact of the mitral leaflet with mobile vegetation on the aortic valve causes inherent weakening of the valve tissue.^10^

HIV‐infected patients have an inherent risk for developing cardiovascular complications. In particular, they are susceptible to atherogenic complications which have been well documented and studied. In 2013, a multicenter HIV‐Heart study (Reinsch et al^11^) evaluated the association between HIV infection with valvular disorders in a prospective, multicenter, cohort study with a population of 803 adults (age 44 ± 10 years, 16.6% women).^11^ The study further demonstrated that clinical stages of HIV infection, as defined by the Center of Disease Control and Prevention (CDC) classification, were associated with higher rates of valvular dysfunction.^11^ Yet, it should be emphasized that there was no association between CD4 cell count and virus load in determining valvular pathology.^11^ It is believed that patients with a suppressed immune system are more susceptible to chronic infections, but HIV‐infected patients also tend to consume intravenous drugs both of which may lead to valvular pathology.^11^ In our patient, serological findings showed a viral load of 49 000 copies per mL of blood and a CD4+ count of 233 cells/mm^3^. Clinical assessment demonstrated that the patient had persistent generalized lymphadenopathy with no evidence of prior AIDS‐defining illnesses, thus making the patient CDC stage A2. As per the 2013 HIV‐Heart study, we would expect our patient to have mild tricuspid lesions per his CDC staging and IV drug history. However, the patient's chronic history of HIV and years of noncompliance with antiretrovirals/follow‐up likely contributed to the diagnosis of moderate mitral regurgitation, which when compared to the 2013 HIV‐Heart study, showed up in only 2.0% of their HIV‐infected patients.

The size of the AML perforation, measuring at 1.77 cm^2^ in this case, is consistent with that found in literature. In a retrospective review by the Mayo Clinic (2006), the size of AML perforations in patients with MVR varied from 0.25 to 4.0 cm^2^. Although that particular study was limited in that only 26 patients participated, it reveals the large range of perforation sizes that can exist. In addition, 6 of the 26 patients in that study had associated vegetations found on echocardiography.^2^ While the lack of vegetations detected on this patient's echocardiography is not unusual, it does add to the subclinical picture of his disease.

In terms of surgical management, prior studies indicate that large defects can be successfully patched with autologous pericardium or polytetrafluoroethylene, while smaller defects of the AML can be successfully closed with 4‐0 or 5‐0 prolene polypropylene sutures.^2^ It should be noted, however, that MV replacement carries the burden of prosthetic valve‐related complications such as bleeding, valve deterioration, and prosthetic endocarditis.^2^ The age of the patient should be considered when deciding if MV replacement is appropriate, as younger patients will carry the risk longer.^2^ The most important preoperative predictor of prosthetic valve endocarditis was active endocarditis at the time of the operation, and it has been shown that late recurrent endocarditis is reported to be 7%‐14%.^2^ The current recommendation for AML perforation of endocarditis etiology is for valve repair as opposed to valve replacement. This has shown, in long‐term studies, to have a better prognosis in patients.^12^ Because the patient in this case was not an acceptable surgical candidate for valve repair, the surgeon found it more appropriate to replace the valve. Regardless of management choice, surgical attention is required for AML perforations. Overall, this case highlights a subclinical cause of mitral regurgitation that requires a high index of suspicion, appropriate workup, and surgical intervention to diagnose and manage effectively.

## CONFLICT OF INTEREST

None declared.

## AUTHORSHIP

PP: wrote and drafted the manuscript, and discussed the importance of echocardiography for educational merit with Dr. Benz. AA and KL: edited manuscript for accuracy and completeness, assisted with literature search, and discussed the importance of TEE and echocardiography for educational merit with Dr. Benz. MB: contributed as research advisor and provided guidance in submission, and provided insight on cardiac physiology. AT: reviewed and approved manuscript as per CarePoint Health Christ Hospital's patient safety and policies.
